# *Jollab*: A Safe Remedy for Functional Dyspepsia in Persian Medicine

**DOI:** 10.34172/apb.2021.001

**Published:** 2020-11-07

**Authors:** Mehdi Pasalar, Babak Daneshfard, Kamran Bagheri Lankarani

**Affiliations:** ^1^Research Center for Traditional Medicine and History of Medicine, Shiraz University of Medical Sciences, Shiraz, Iran.; ^2^Essence of Parsiyan Wisdom Institute, Phytopharmaceutical Technology and Traditional Medicine Incubator, Shiraz University of Medical Sciences, Shiraz, Iran.; ^3^Traditional Medicine Clinical Trial Research Center, Shahed University, Tehran, Iran.; ^4^Health Policy Research Center, Shiraz University of Medical Sciences, Shiraz, Iran.

## Dear Editor,

With lots of interest, we read the paper by Ebrahimzadeh Attari et al.^1^ In this valuable investigation, the beneficial effects of ginger in eradication of *Helicobacter pylori* and improvement of functional dyspepsia symptoms have been revealed.

Functional dyspepsia (FD) is a common annoying disorder without any approved medication for its treatment; although, some “off-label” treatments including antidepressants are commonly used.^2^

The mind-gut relation and possible beneficial effect of antidepressants in FD has been the subject of several studies. However, these drugs are not completely safe and may induce significant side effects especially in the older patients.^3^ Since a long time ago, old comprehensive medical schools including Ayurveda, Traditional Chinese Medicine, and Persian Medicine (PM) have acknowledged this mind-gut relation through their holistic viewpoints toward health and human well-being. For instance, Avicenna (980–1037 AD) who is a well-known Persian sage of Islamic Golden Age,^4^ has mentioned the importance of psychiatric components in the emergence of FD symptoms in the “*Canon of Medicine* ”. In this regard, the proposed management of dyspepsia in PM, similar to other holistic medical schools, is not only toward the gastrointestinal tract, but also consider the interventions targeting the mind and psychological affairs.^5,6^

We have recently shown that *Jollab*, a mixture of rose water, saffron and candy sugar in syrup dosage form, could improve the FD symptoms as well as the quality of life and depressive symptoms, simultaneously. The compound had good safety profile without any hazardous side effects.^7^ Considering the high rate of adverse events related to antidepressant usage in treating FD and high rate of discontinuation of these drugs in several studies,^3^ the safety profile of *Jollab* is quite interesting. Although believed credence of herbal products’ safety has been failed repeatedly, precise approach to complementary/traditional remedies whit anti-psychotic potential could propose an excellent opportunity for handling the prevalent diseases such as FD. This could also diminish the side effects and improve the patients’ satisfaction.

Unfortunately, data on the use of PM medicinal herbs are sparse and many gastroenterologists even in Iran are not familiar with this approach. However, in the current era of *integrative medicine*, such approaches should receive more attention from the physicians.

## Ethical Issues

Not applicable.

## Conflict of Interest

There is no conflict of interest to declare.
